# Involved-Field Radiotherapy versus Elective Nodal Irradiation in Combination with Concurrent Chemotherapy for Locally Advanced Non-Small Cell Lung Cancer: A Prospective Randomized Study

**DOI:** 10.1155/2013/371819

**Published:** 2013-05-13

**Authors:** Ming Chen, Yong Bao, Hong-Lian Ma, Xiao Hu, Jin Wang, Yan Wang, Fang Peng, Qi-Chao Zhou, Cong-Hua Xie

**Affiliations:** ^1^Department of Radiation and Medical Oncology, Zhongnan Hospital, Hubei Cancer Clinical Study Center, Wuhan University, Wuhan 430071, China; ^2^Department of Radiation Oncology, Sun Yat-sen University Cancer Center, State Key Laboratory of Oncology in South China, Guangzhou 510060, China; ^3^Department of Radiation Oncology, Zhejiang Cancer Hospital, Hangzhou 310022, China

## Abstract

This prospective randomized study is to evaluate the locoregional failure and its impact on survival by comparing involved field radiotherapy (IFRT) with elective nodal irradiation (ENI) in combination with concurrent chemotherapy for locally advanced non-small cell lung cancer. It appears that higher dose could be delivered in IFRT arm than that in ENI arm, and IFRT did not increase the risk of initially uninvolved or isolated nodal failures. Both a tendency of improved locoregional progression-free survival and a significant increased overall survival rate are in favor of IFRT arm in this study.

## 1. Introduction

Nowadays the combined chemoradiotherapy has been established as a standard treatment modality for patients with unresectable locally advanced non-small cell lung cancer (LA-NSCLC). However, overall survival remains poor with median survival time 16–20 months because of both local and distant failure [1]. Studies have confirmed that improved local control and overall survival are associated with dose escalation in patients with LA-NSCLC. But the use of traditional elective nodal irradiation (ENI) will limit dose escalation because of pulmonary and esophageal toxicities [2]. So some investigators used involved-field radiotherapy (IFRT) in order to accomplish dose escalation [3–5]. However, rare data from large sample sizes, prospective, randomized studies were available to support the use of IFRT. Whether IFRT could replace ENI or not has been a controversial topic for years [6]. Therefore, we have initiated this prospective randomized study on IFRT versus ENI in combination with concurrent chemotherapy for LA-NSCLC since July 2002, with a primary objective of the locoregional progression and failure patterns, as well as the impact of failure patterns on overall survival (OS).

## 2. Materials and Methods

### 2.1. General Clinical Data

Inclusion criteria were as follows. Patients were eligible when histologic or cytologic of NSCLC verified and stage IIIA or IIIB confirmed radiographically (according to the 6th AJCC/UICC staging system) with no pleural effusion (including brain magnetic resonance imaging, contrast-enhanced chest and abdomen CT, and bone scintigraphy, while positron-emission tomography was not mandatory). The patients were aged 18–75 years without previous thoracic radiotherapy or chemotherapy. Karnofsky performance status was ⩾70 and had measurable or assessable disease, neutrophilic granulocyte ⩾1.5 × 10^9^/L, hemoglobin ⩾100 g/L, and platelet count ⩾100 × 10^9^/L. Serum creatine and bilirubin were <1.5 × the upper normal limit (UNL). Aminotransferase was <2 × UNL. Weight loss was less than 10% within 6 months before diagnosis. Written informed consent was required from all patients. 

Exclusion criteria were as follows. Patients were ineligible when they had a history of other malignant diseases except for non-melanomatous skin cancer, carcinoma in situ of the cervix, or any contraindications for chemoradiotherapy, malignant pleural, and/or pericardial effusion.

### 2.2. Interventions

*Chemotherapy*. Patients were randomized into IFRT arm or ENI arm. Induction chemotherapy consisted of paclitaxel (175 mg/m^2^ on day 1) and carboplatin (AUC = 5*∼*6 on day 1) administered intravenously at 21-day intervals for 2 cycles. Three to four weeks after induction chemotherapy, thoracic radiotherapy was administered concurrently with weekly paclitaxel (40 mg/m^2^) as a radiosensitizer to end radiotherapy [7]. During chemotherapy, 5-hydroxytryptamine receptor antagonists, dexamethasone, cimetidine, and diphenhydramine were used prophylactically. Meanwhile, electrocardio-guardianship was applied.

*Radiotherapy*. All patients were immobilized in a supine position on a vacuum bag with both arms above the head; a contrast-enhanced CT simulation was performed from the fourth cervical vertebra to the second lumbar vertebra, using a maximal slice thickness of 5 mm. A three-dimensional treatment planning system of Pinnacle (version 7.0∼8.0) was applied to the radiotherapy plan. Radiotherapy was performed with a linear accelerator using 6∼8 MV photons. After induction chemotherapy, patients who did not develop metastasis continued to receive concurrent chemoradiotherapy. The targets were contoured in accordance with the International Commission on Radiation Units and Measurements (ICRU) 50 guidelines. Gross tumor volume (GTV) included the gross tumor volume-tumor (GTV-T) and gross tumor volume-node (GTV-N) defined as lymph nodes in the ipsilateral hilum and mediastinum with a short diameter ⩾1 cm, or lymph nodes with positive tumor cell sampling, or clusters of small lymph nodes of short diameter <1 cm within one region, or 18F-FDG standard uptake value ⩾2.5 on PET/CT at initial staging. The lymph node regions originally involved before induction chemotherapy were included in the radiation fields for both arms even when the lymph nodes disappeared after induction chemotherapy. The clinical target volume-tumor (CTV-T) included the GTV-T with a margin of 0.6 cm or 0.8 cm, depending on squamous cell carcinoma or otherwise in all patients [8]. For patients who were randomized to IFRT arm, the clinical target volume-node (CTV-N) included the prechemotherapy positive lymph nodes with a margin of 0.5 cm and ipsilateral hilum. For patients who were randomized to ENI arm, the CTV-N included the ipsilateral hilum, mediastinum (from the inferior head of the clavicle to 3*∼*5 cm below the carina), and the bilateral supraclavicular fossa. CTVs (include CTV-T and CTV-N) were edited according to the anatomic structure borders. Planning target volumes (PTV) involved CTVs with a margin of 1.0 cm*∼*1.5 cm. Under the condition of V20 (percent volume of bilateral lung receiving ⩾20 Gy) ⩽35% and the maximal dose to spinal cord ⩽50 Gy, thoracic irradiation consisted of 2.0 Gy once a day for consecutive 5 days a week to a maximal tolerable dose in IFRT arm. In ENI arm, a dose of 40*∼*46 Gy was delivered to the elective nodal areas. Then an escalated dose was delivered to GTV to a maximal tolerable dose. 

### 2.3. Follow-Up

Patients took chest X-ray exam every 2 weeks during irradiation period. After completion of treatment, patients were reviewed within 4*∼*6 weeks, then every 3 months in the first 2 years, every 4 months in the third year, and every 6 months thereafter. Physical examination and CT scans of the thorax and upper abdomen were performed routinely. 

### 2.4. Response and Toxicity Criteria

Tumor response was evaluated with thoracic CT scans after induction chemotherapy and concurrent chemoradiotherapy were completed, in accordance with Response Evaluation Criteria in Solid Tumors Group 1.0 (RECIST1.0). An elective nodal failure (ENF) was defined as a nodal failure of elective irradiation region in ENI arm and an uninvolved nodal failure out of irradiation field in IFRT arm. Involved-field nodal failure (IFNF) was defined as a nodal failure in the irradiation region with dose escalation in ENI arm and a nodal failure in irradiation field in IFRT arm. Locoregional failure included primary tumor failure, ENF and IFNF. Local progression-free survival (LPFS) was recorded from the beginning of induction chemotherapy to the time of primary tumor failure, ENF or IFNF. During radiotherapy, acute radiation-induced pneumonitis and esophagitis as well as body weight change of each patient was recorded, and a complete blood count was performed at least once a week. Acute hematologic toxicities and weight loss were classified in accordance with the National Cancer Institute Common Toxicity Criteria (CTCAE) version 3.0. Acute and late toxicities of lung and esophagus were evaluated according to RTOG criteria [9]. 

### 2.5. Study Design and Statistical Analysis

This study was designed as a prospective, randomized trial. The primary endpoint was locoregional progression. We hypothesized that the 3-year local control rates for IFRT arm and ENI arm were 45% and 30%, respectively; sample sizes of 123 in each group achieved 80% power to detect a risk ratio of 0.65. A statistical software package SPSS 15.0 (IBM, Somers, New York) was applied, and the Kaplan-Meier method was used to estimate survival data. The distribution of survival time between arms was tested by log-rank method; a Student *t*-test was used for comparison of means. Fisher exact test was used for comparisons of categorical data. All *P* values were based on a 2-sided test, and the differences were regarded as statistically significant when *P* < 0.05. The protocol was approved by the clinical ethics committee of Sun Yat Sen University Cancer Center before study activation.

## 3. Results

### 3.1. Patient Characteristics

Between July 2002 and June 2011, a total of consecutive 99 patients with LA-NSCLC were enrolled onto the study. The characteristics of the 99 eligible patients were well balanced in 2 arms (Table 1). Eight patients were ineligible because of distant metastasis during chemoradiotherapy and were not included for local control analysis.

### 3.2. Treatment Results

Forty and 48 patients had completed radiation plan in IFRT arm and ENI arm, respectively. Eight patients developed distant metastasis during radiotherapy received palliative treatment. Of the 3 patients who discontinued radiation plan, one emerged acute left ventricular failure when irradiated to 40 Gy in IFRT arm, one developed severe acute respiratory syndromes when irradiated to 46 Gy, and one refused treatment because of grade 2 radiation-induced esophagitis when irradiated to 32 Gy in ENI arm. The media radiation dose were 60 Gy (range: 38*∼*74 Gy) and 60 Gy (range: 32*∼*70 Gy) in IFRT arm and ENI arm, respectively. Thirty-six (87.8%) and 40 (80.0%) patients received dose of ⩾60 Gy, respectively (*P* = 0.426). More patients in IFRT arm received ⩾62 Gy than those in ENI arm (48.9% versus 25.9%, *P* = 0.018). Rank sum test was used for comparing dose distribution between the two arms. Dose delivered in IFRT arm was higher than that in ENI arm. The mean rank order was 51.84 and 41.21 in IFRT arm and ENI arm, respectively (*P* = 0.042). The average total cycles of concurrent chemotherapy administered in IFRT arm and ENI arm were 5.5 ± 1.4 and 5.9 ± 1.1, respectively (*P* = 0.168). 

### 3.3. Locoregional Failure, Distant Metastasis, and Survival

Patients who developed distant metastasis after induction chemotherapy were not included in analysis for locoregional but for distant failure. At last follow-up, 14 (34.1%) patients in IFRT arm and 15 (30%) patients in ENI arm experienced locoregional failure (*P* = 0.673). Among them, 9 (22%) and 12 (24%) encountered primary tumor failure in IFRT arm and in ENI arm, respectively. ENF in combination with involved-field nodal failure (IFNF) or primary recurrence was present in 2 and 3 patients, respectively. Only one patient experienced isolated-ENF in ENI arm. Twenty-four (53.3%) patients in IFRT arm and 31 (57.4%) cases in ENI arm experienced distant metastases.

**Figure 1:**
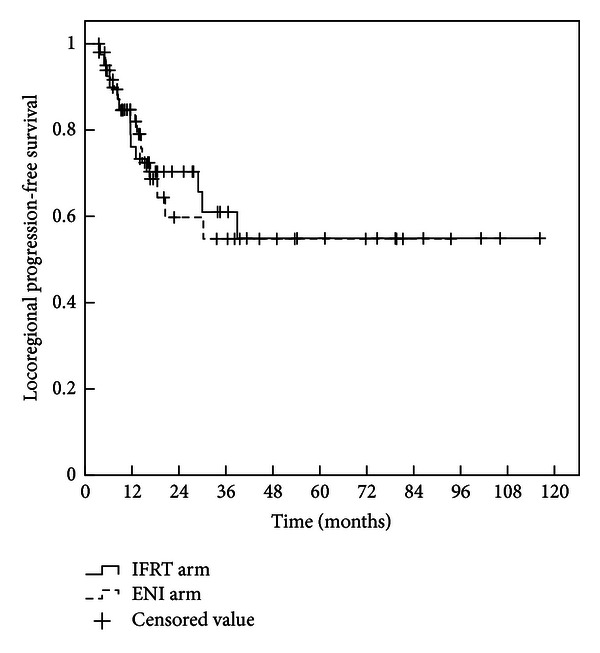
Local progression-free survival curves for patients with IFRT or ENI. The 1-, 2-, and 3-year local tumor progression-free survival rates in the IFRT arm were 78.1%, 72.6%, and 62.9% compared with 85.5%, 61.2%, and 56.1%, respectively, in the ENI arm. There was no statistically significant difference in local progression-free survival between the two arms (*P* = 0.895).

**Figure 2:**
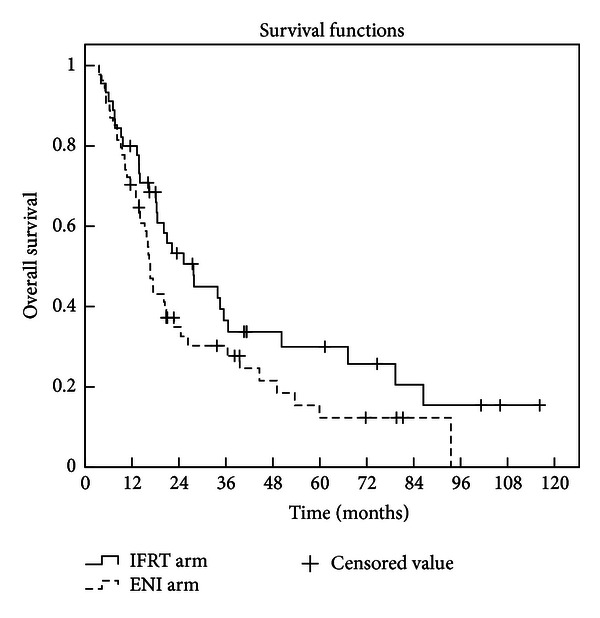
Overall survival curves for patients with IFRT or ENI. The 1-, 2-, and 3-year overall survival rates in the IFRT arm were 80.0%, 53.3%, and 36.6% compared with 70.4%, 34.9%, and 30.3%, respectively, in the ENI arm. There was no statistically significant difference in local progression-free survival between the two arms (*P* = 0.08).

**Figure 3:**
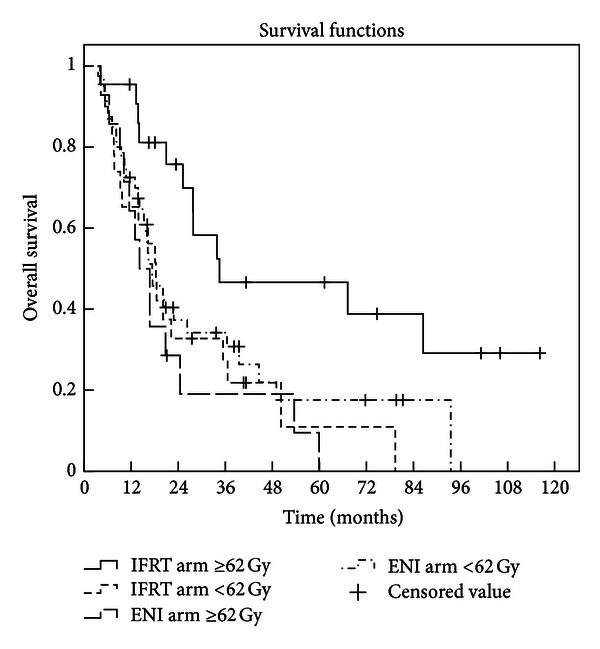
Overall survival of patients irradiated at dose of ⩾62 Gy in IFRT arm, much better than that of those patients who received radiation dose <62 Gy in both arms and that when radiation dose was greater than or equal to 62 Gy in ENI arm (*P* = 0.013).

Twenty-five patients remained alive at the time of analysis, with a median follow-up of 33.6 months in survivors (4.8 months*∼*112 months). The median LPFS time was not available. The 1-, 2-, and 3-year LPFS rates were 78.1%, 72.6%, and 62.9%, respectively, in IFRT arm, versus 85.5%, 61.2.0%, and 56.1% in ENI arm (*P* = 0.895 by log-rank test) as shown in Figure 1. The median survival time was 27.8 months in IFRT arm (95% confidence interval (CI), 18.0*∼*37.5 months) and 16.7 months (95% CI, 15.0*∼*18.4 months) in ENI arm. The 1-, 2-, and 3-year OS rates were 80.0%, 53.3%, and 36.6%, respectively, in IFRT arm, versus 70.4%, 34.9%, and 30.3% in ENI arm (*P* = 0.08 by log-rank test) as shown in Figure 2. The 1-, 2-, and 3-year OS rates were 95.5%, 75.7%, and 46.6%, respectively, in IFRT arm when radiation dose ⩾62 Gy, much better than that of those patients who received radiation dose <62 Gy in both arms, and that in ENI arm when radiation dose was greater than or equal to 62 Gy (*P* = 0.013 by log-rank test) as shown in Figure 3.

### 3.4. Toxicity

(i) *Acute Toxicities.* Hematologic and nonhematologic acute and late toxicities are summarized in Table 2. Hematologic toxicity was mild to moderate in both arms; one patient developed grade III radiation-induced pneumonitis in each arm and 1 patient encountered grade IV radiation-induced pneumonitis in IFRT arm. No severe esophageal toxicity was observed in two arms. There were no significant differences in acute nonhematologic toxicities between the arms. 

(ii) *Late Toxicities.* Late toxicities of radiotherapy were mainly mild-to-moderate pulmonary and esophageal injury. No patients developed grade III*∼*IV pulmonary injury in both arms. Late spinal cord toxicity was not observed. Our study showed no significant differences in toxicity in patients treated with IFRT or ENI using three-dimensional conformal radiotherapy (3D-CRT) technique for LA-NSCLC.

## 4. Discussion

In order to avoid missing the targets, ENI was applied and relatively decreased the failure rate of mediastinal lymph nodes in 2-dimensional radiotherapy era. However, with the advent of three-dimensional conformal radiotherapy (3D-CRT), ENI considerably increased exposure of normal tissues, led to significant toxicities, and prevented dose-escalation. RTOG 9311 [10] was a phase I*∼*II dose-escalation study for patients with inoperable NSCLC treated with 3D-CRT. Elective nodal regions were not intentionally irradiated. The radiation dose was safely escalated to 77.4 Gy when V20 was between 25% and 36% and to 83.8 Gy when V20 <25%. The 2-year locoregional control rate was 50%*∼*78%. Although postoperative pathologic upstaging to N2 was reported in approximately 20% of patients with presurgery diagnosis as N0/1 [11, 12], micrometastases were detected in 20.4% of patients with pathologic N0 when immunohistochemical staining was used [13]. The isolated-ENF rate of 7% was reported in Stage T1-2N0 M0 NSCLC patients treated with stereotactic body radiation therapy (SBRT) [14]. Several studies have reported that the ENF rate was less than 10% in patients with NSCLC treated with IFRT [3, 5, 15–18].

Fernandes et al. [19] retrospectively analyzed 108 patients with locally advanced NSCLC treated with 3DCRT and concurrent or sequential chemotherapy. The IFRT group received higher radiation doses than the ENI group (60*∼*84 Gy versus 54*∼*72 Gy, *P* < 0.001). There were no significantly differences in ENF rate between the 2 groups. To our knowledge, there are few prospective studies available comparing ENI with IFRT in patients with LA-NSCLC at present. Only one randomized trial specifically addressed this issue [20]. In this trial, 68*∼*74 Gy radiation doses were prescribed for patients in IFRT arm, while 60∼64 Gy for the ENI arm. ENF rate was 7% and 4% in IFRT arm and ENI arm, respectively (*P* = 0.352). Patients in the IFRT arm achieved better 5-year local control (LC) rate (51% versus 36%, *P* = 0.032) and better 2-year survival rate (39.4% versus 25.6%, *P* = 0.048) than those in the ENI arm. 

Because the prescribed dose to IFRT arm was higher than that to ENI arm, it remained unclear if the better outcome from IFRT was due to the higher radiation dose or the use of IFRT in the study above. Therefore, our study is designed uniquely in that we delivered radiation dose as high as possible provided that the organs at risk could tolerate. Under the condition of that V20 was ⩽35% and the maximum dose to spinal cord was ⩽50 Gy, dose delivered between the IFRT arm and ENI arm was significantly different. Patients in IFRT arm received dose of ⩾62 Gy which was much more than that in ENI arm (48.9% versus 25.9%, *P* = 0.018), which indicated that with IFRT, higher dose could be delivered. It was the same as the results of many studies on radiation dose escalation [4, 10, 18, 21]. 

Sura et al. [22] discovered that when the dose delivered was <60 Gy or ⩾60 Gy in NSCLC patients treated with IFRT, 75% patients and 33% patients developed recurrence within the GTV, respectively (*P* < 0.05). Rengan et al. [23] reported that the 1- and 2-year local failure rates were 27%, 47% and 61%, 76%, respectively, for stage III patients treated with 64 Gy or higher, and less than 64 Gy (*P* = 0.024). Both studies suggested that administration of higher doses using 3D-CRT improved local control in NSCLC patients. In our study, only one patient developed isolated-ENF in ENI arm. Two patients in IFRT arm and 3 in ENI arm had ENF accompanied with IFNF or primary tumor failure. Less patients developed ENF in IFRT arm than in ENI arm (4.9% versus 8.0%), which suggests that a decrease in ENF may be achieved when involved-field control rate increased due to a higher prescription dose delivered in the IFRT arm. The preliminary results have showed that the median overall survival in IFRT arm was longer than that in ENI arm (27.8 months versus 16.7 months). In addition, the overall survival rate of patients treated with IFRT at dose of ⩾62 Gy is significantly higher than that in both arms at dose of <62 Gy and that in the ENI arm at dose of ⩾62 Gy as showed in Figure 3 (*P* = 0.013). These results indicate that irradiation dose could be successfully escalated with IFRT and then overall survival rate is expected to be increased. 

However, at the 53rd Annual Meeting of the American Society of Radiation Oncology, Bradley et al. [24] reported the result of RTOG 0617 study that the higher radiation dose of 74 Gy could not produce an overall survival benefit compared with 60 Gy. These findings are counterintuitive and run counter to a large body of evidence showing that higher radiation doses lead to better tumor control at numerous sites. Moreover, the toxicity rates difference was not significant statistically between the two dose groups. However, there were 17 patients died in the 74-Gy arms and 7 in the 60-Gy arms. Cox [25] reviewed considerable evidence that supports the hypothesis that the pulmonary or cardiopulmonary effects of thoracic radiotherapy can contribute to death and considered that as the most likely explanation of the findings from the RTOG 0617. So we could still presume that if the death-related toxicities can be restrained in higher dose arms, dose escalation remains produce survival benefit. In our study, dose to organs at risk was restrained in both arms, so there was no significant deference in toxicities between the two arms. Even if the final result of RTOG 0617 study would confirm that higher radiation dose could not improve survival, delivered with same dose, irradiation with IFRT could decrease normal tissues damage due to less normal tissues irradiation exposure than that with ENI. Then treatment prevalence would be increased and a survival benefit even to be produced.

In summary, our preliminary results showed that IFRT did not increase the risk of initially uninvolved or isolated nodal failures and locoregional failure. Meanwhile, higher radiation dose could be effectively administered with IFRT, which is expected to improve overall survival. 

The current sample size has not met the designed requirements; caution must be taken when adopting the conclusions. Further investigations are warranted. In addition, CT scan was used to assess the treatment effect and failure patterns for all patients. Undetected occult recurrence and micrometastasis of mediastinal lymph node maybe exist as the intrinsic limitation of accuracy using CT scan [26]. Nowadays, PET/CT scans are available generally, which is superior to CT for assessment of stage III NSCLC after chemoradiation [27]. In future studies, we hope to ideally incorporate information from PET/CT scans for diagnosis and assessment.

## 5. Conclusions 

These preliminary results indicated that IFRT did not increase locoregional failure related to ENF. With IFRT, higher radiation dose could be administered compared with ENI and it is expected to improve survival. Further investigation is warranted.

## Figures and Tables

**Table 1:** Patient characteristics.

Characteristic	IFRT arm (*n* = 45)		ENI arm (*n* = 54)		*P *
	*n*	%	*n*	%	
Gender					0.343
Male	37	82.2	48	88.9	
Female	8	17.8	6	11.1	
Age, y					0.385
Median	56		55.5		
Range	27~71		38~71		
KPS					0.744
80–90	8	17.8	11	20.4	
90–100	37	82.2	43	79.6	
Weight loss					0.771
<5%	34	75.6	44	81.5	
5%–10%	10	22.2	9	16.7	
>10%	1	2.2	1	1.9	
TNM stage					0.420
IIIA	15	33.3	14	25.9	
IIIB	30	66.7	40	74.1	
Tumor position					0.363
Central	33	73.3	35	64.8	
Peripheral	12	26.7	19	35.2	
PET/CT examination	13	28.9	10	18.5	0.224
Histology					0.668
Adenocarcinoma	23	51.1	27	50.0	
Squamous cell carcinoma	19	42.2	23	42.6	
Adenosquamous carcinoma	1	2.2	0	0.0	
Undifferentiated carcinoma	2	4.4	4	7.4	

**Table 2:** Treatment toxicities according to treatment arms.

	IFRT arm (*n* = 41)		ENI arm (*n* = 50)		*P *
	*n*	%	*n*	%	
Acute toxicities					
Hematologic toxicity ≥grade 3					
Leucopenia					0.384
III	31	75.6	38	76.0	
IV	3	7.3	2	4.0	
Anemia					0.499
III	1	2.4	0	0.0	
IV	1	2.4	0	0.0	
Weight loss					0.256
I	8	19.5	4	8.0	
II	2	4.9	2	4.0	
Pneumonitis					0.385
I-II	23	56.1	22	44.0	
III-IV	2	4.8	1	2.0	
Esophagitis					0.839
0-I	27	65.9	35	70.0	
II	14	34.1	15	30.0	
Late toxicities					
Pulmonary injury					0.925
I-II	19	46.3	23	46.0	
III–V	0	0.0	0	0.0	
Esophageal injury					0.142
I-II	3	7.3	4	8.0	
III-IV	0	0.0	0	0.0	

## References

[1] Vokes EE, Herndon JE, Kelley MJ (2007). Induction chemotherapy followed by chemoradiotherapy compared with chemoradiotherapy alone for regionally advanced unresectable stage III non-small-cell lung cancer: cancer and leukemia group B. *Journal of Clinical Oncology*.

[2] Bradley JD, Wahab S, Lockett MA, Perez CA, Purdy JA (2003). Elective nodal failures are uncommon in medically inoperable patients with Stage I non-small-cell lung carcinoma treated with limited radiotherapy fields. *International Journal of Radiation Oncology ∗ Biology ∗ Physics*.

[3] Rosenzweig KE, Sura S, Jackson A, Yorke E (2007). Involved-field radiation therapy for inoperable non-small-cell lung cancer. *Journal of Clinical Oncology*.

[4] Hayman JA, Martel MK, Ten Haken RK (2001). Dose escalation in non-small-cell lung cancer using three-dimensional conformal radiation therapy: update of a phase I trial. *Journal of Clinical Oncology*.

[5] Kong FM, Ten Haken RK, Schipper MJ (2005). High-dose radiation improved local tumor control and overall survival in patients with inoperable/unresectable non-small-cell lung cancer: long-term results of a radiation dose escalation study. *International Journal of Radiation Oncology ∗ Biology ∗ Physics*.

[6] Belderbos JSA, Kepka L, (Spring) Kong F.-M. FM, Martel MK, Videtic GMM, Jeremic B (2008). Report from the International Atomic Energy Agency (IAEA) consultants' meeting on elective nodal irradiation in lung cancer: non-small-cell lung cancer (NSCLC). *International Journal of Radiation Oncology ∗ Biology ∗ Physics*.

[7] Choy H, Safran H, Akerley W, Graziano SL, Bogart JA, Cole BF (1998). Phase II trial of weekly paclitaxel and concurrent radiation therapy for locally advanced non-small cell lung cancer. *Clinical Cancer Research*.

[8] Giraud P, Antoine M, Larrouy A (2000). Evaluation of microscopic tumor extension in non-small-cell lung cancer for three-dimensional conformal radiotherapy planning. *International Journal of Radiation Oncology ∗ Biology ∗ Physics*.

[9] Cox JD, Stetz J, Pajak TF (1995). Toxicity criteria of the Radiation Therapy Oncology Group (RTOG) and the European organization for research and treatment of cancer (EORTC). *International Journal of Radiation Oncology ∗ Biology ∗ Physics*.

[10] Bradley J, Graham MV, Winter K (2005). Toxicity and outcome results of RTOG 9311: a phase I-II dose-escalation study using three-dimensional conformal radiotherapy in patients with inoperable non-small-cell lung carcinoma. *International Journal of Radiation Oncology ∗ Biology ∗ Physics*.

[11] Graham ANJ, Chan KJM, Pastorino U, Goldstraw P (1999). Systematic nodal dissection in the intrathoracic staging of patients with non-small cell lung cancer. *Journal of Thoracic and Cardiovascular Surgery*.

[12] Koike T, Terashima M, Takizawa T, Watanabe T, Kurita Y, Yokoyama A (1998). Clinical analysis of small-sized peripheral lung cancer. *Journal of Thoracic and Cardiovascular Surgery*.

[13] Wu J, Ohta Y, Minato H (2001). Nodal occult metastasis in patients with peripheral lung adenocarcinoma of 2.0 cm or less in diameter. *Annals of Thoracic Surgery*.

[14] Andratschke N, Zimmermann F, Boehm E, Schill S, Schoenknecht C (2011). Stereotactic radiotherapy of histologically proven inoperable stage I non-small cell lung cancer: patterns of failure. *Radiotherapy and Oncology*.

[15] Sulman EP, Komaki R, Klopp AH, Cox JD, Chang JY (2009). Exclusion of elective nodal irradiation is associated with minimal elective nodal failure in non-small cell lung cancer. *Radiation Oncology*.

[16] De Ruysscher D, Wanders S, van Haren E (2005). Selective mediastinal node irradiation based on FDG-PET scan data in patients with non-small-cell lung cancer: a prospective clinical study. *International Journal of Radiation Oncology ∗ Biology ∗ Physics*.

[17] Rosenzweig KE, Sim SE, Mychalczak B, Braban LE, Schindelheim R, Leibel SA (2001). Elective nodal irradiation in the treatment of non-small-cell lung cancer with three-dimensional conformal radiation therapy. *International Journal of Radiation Oncology ∗ Biology ∗ Physics*.

[18] Belderbos JSA, Heemsbergen WD, De Jaeger K, Baas P, Lebesque JV (2006). Final results of a phase I/II dose escalation trial in non-small-cell lung cancer using three-dimensional conformal radiotherapy. *International Journal of Radiation Oncology ∗ Biology ∗ Physics*.

[19] Fernandes AT, Shen J, Finlay J (2010). Elective nodal irradiation (ENI) versus involved field radiotherapy (IFRT) for locally advanced non-small cell lung cancer (NSCLC): a comparative analysis of toxicities and clinical outcomes. *Radiotherapy and Oncology*.

[20] Yuan S, Sun X, Li M (2007). A randomized study of involved-field irradiation versus elective nodal irradiation in combination with concurrent chemotherapy for inoperable stage III nonsmall cell lung cancer. *American Journal of Clinical Oncology*.

[21] Belderbos JSA, De Jaeger K, Heemsbergen WD (2003). First results of a phase I/II dose escalation trial in non-small cell lung cancer using three-dimensional conformal radiotherapy. *Radiotherapy and Oncology*.

[22] Sura S, Greco C, Gelblum D, Yorke ED, Jackson A, Rosenzweig KE (2008). (18)F-fluorodeoxyglucose positron emission tomography-based assessment of local failure patterns in non-small-cell lung cancer treated with definitive radiotherapy. *International Journal of Radiation Oncology ∗ Biology ∗ Physics*.

[23] Rengan R, Rosenzweig KE, Venkatraman E (2004). Improved local control with higher doses of radiation in large-volume stage III non-small-cell lung cancer. *International Journal of Radiation Oncology ∗ Biology ∗ Physics*.

[24] Bradley JD, Paulus R, Komaki R A randomized phase III comparison of standard-dose (60 Gy) versus high-dose (74 Gy) conformal chemoradiotherapy +/- cetuximab for stage IIIA/IIIB non-small cell lung cancer: preliminary findings on radiation dose in RTOG 0617.

[25] Cox JD (2012). Are the results of RTOG, 0617 mysterious?. *International Journal of Radiation Oncology ∗ Biology ∗ Physics*.

[26] Toloza EM, Harpole L, McCrory DC (2003). Noninvasive staging of non-small cell lung cancer: a review of the current evidence. *Chest*.

[27] Cerfolio RJ, Bryant AS, Ojha B (2006). Restaging patients with N2 (stage IIIa) non-small cell lung cancer after neoadjuvant chemoradiotherapy: a prospective study. *Journal of Thoracic and Cardiovascular Surgery*.

